# Association of Diabetes and Tuberculosis Disease among US-Bound Adult Refugees, 2009–2014

**DOI:** 10.3201/eid2303.161053

**Published:** 2017-03

**Authors:** Stephen R. Benoit, Edward W. Gregg, Sasi Jonnalagadda, Christina R. Phares, Weigong Zhou, John A. Painter

**Affiliations:** Centers for Disease Control and Prevention, Atlanta, Georgia, USA

**Keywords:** tuberculosis, tuberculosis and other mycobacteria, diabetes, refugees, bacteria

## Abstract

Diabetes is associated with an increased risk for active tuberculosis (TB) disease. We conducted a case–control study and found a significant association between diabetes and TB disease among US-bound refugees. These findings underscore the value of collaborative management of both diseases.

The burden of tuberculosis (TB) is highest in resource-limited countries, many of which are experiencing increased rates of diabetes (*1*,*2*). Because of the effect of diabetes on the immune system, risk for active TB disease is higher and treatment outcomes are poorer among persons with diabetes (*3*). In 2011, the World Health Organization and the International Union Against Tuberculosis and Lung Disease developed a framework for a coordinated response to both diseases and advised screening all TB patients for diabetes (*4*).

Annually, ≈70,000 refugees resettle in the United States (*5*). Before departure, all refugees undergo a medical examination and screening for conditions of public health importance; TB is considered a priority condition (*6*). The Centers for Disease Control and Prevention Electronic Disease Notification (EDN) System captures data from these examinations (*7*). Domestic screening examinations are recommended within 3 months of arrival in the United States, and the TB component is captured in EDN.

Using EDN, we examined the association of diabetes and TB disease in our target population, which consisted of 249,037 US-bound refugees >18 years of age at the time of their overseas medical examination who arrived in the United States from January 1, 2009, through August 31, 2014. We excluded 187 records because of missing data. TB disease was defined as clinical or laboratory-diagnosed disease, either 1) active pulmonary or extrapulmonary TB diagnosed during the overseas examination prior to departure and treated before arrival in the United States (*6*) or 2) diagnosis of active TB at the domestic examination after entry into the Unites States. 

Diabetes screening is not a requirement for admission to the United States. However, if reported by the refugee while recording the medical history or discovered during the overseas examination process, diabetes should be documented on the medical examination forms. Using text parsing techniques described previously (*8*), we searched for evidence of diabetes in these forms.

Demographic variables were sex, age group, living setting (refugee camp or noncamp setting), and region of nationality, which were assigned according to US Department of State categories. Body mass index was categorized as underweight (<18.5 kg/m^2^), normal (18.5 to <25 kg/m^2^), overweight (25 to <30 kg/m^2^), or obese (>30 kg/m^2^). 

We used logistic regression to assess the association of diabetes and TB and assessed effect modification between region and diabetes. Variables were included in the multivariate model if they were significant (p<0.05) in a model of diabetes only or if they were confounders, defined as variables causing a change in odds between diabetes and TB of >20%.

From January 1, 2009, through August 31, 2014, according to our case definitions, 2,262 (0.9%) of 248,850 US-bound refugees >18 years of age had TB, 5,767 (2.3%) had diabetes, and 56 (<0.1%) had both. Effect modification between region and diabetes was not significant. After controlling for region, sex, age group, body mass index, and living in a refugee camp, we found a significant association between diabetes and TB (adjusted odds ratio 1.7, 95% CI 1.3–2.2) (Table).

**Table:** Characteristics of US-bound adult refugees and association of diabetes with TB disease, Electronic Disease Notification System, January 2009–August 2014*

Characteristic	Total no. (%), N = 248,850	TB, no. (%), n = 2,262	Odds ratio (95% CI)	
			Univariate	Multivariate
Diabetes	5,767 (2.3)	56 (2.5)	1.1 (0.8–1.4)	1.7 (1.3–2.2)
Region				
Africa	40,731 (16.4)	422 (18.7)	Reference	Reference
East Asia and the Pacific	58,701 (23.6)	971 (42.9)	1.6 (1.4–1.8)	1.7 (1.5–1.9)
Europe and Eurasia	3,867 (1.6)	22 (1.0)	0.5 (0.4–0.8)	0.6 (0.4–1.0)
Near East	76,752 (30.8)	44 (2.0)	0.1 (<0.1–0.1)	0.1 (0.1–0.1)
South and Central Asia	52,677 (21.2)	802 (35.5)	1.5 (1.3–1.7)	1.2 (1.0–1.4)
Western Hemisphere	16,122 (6.5)	1 (<0.1)	<0.1 (<0.1–<0.1)	<0.1 (<0.1–<0.1)
Female sex	115,297 (46.3)	747 (33.0)	0.6 (0.5–0.6)	0.6 (0.5–0.6)
Age group, y				
18–44	190,141 (76.4)	1,391 (61.5)	Reference	Reference
45–64	45,711 (18.4)	600 (26.5)	1.8 (1.6–2.0)	2.6 (2.3–2.8)
65–74	9,397 (3.8)	175 (7.7)	2.6 (2.2–3.0)	3.6 (3.0–4.3)
>75	3,601 (1.5)	96 (4.2)	3.7 (3.0–4.6)	5.0 (4.0–6.3)
BMI category†				
Underweight	20,300 (8.6)	390 (18.3)	1.7 (1.5–1.9)	1.6 (1.4–1.8)
Normal	127,033 (53.7)	1,465 (68.8)	Reference	Reference
Overweight	58,299 (24.7)	233 (10.9)	0.3 (0.3–0.4)	0.5 (0.5–0.6)
Obese	30,731 (13.0)	43 (2.0)	0.1 (0.1–0.2)	0.4 (0.3–0.5)
Lived in refugee camp‡	88,490 (36.0)	1,448 (64.6)	3.3 (3.0–3.6)	1.2 (1.0–1.3)

*Adults were those >18 years of age. BMI, body mass index; TB, tuberculosis. †Proportions based on nonmissing data; 5.0% missing data. ‡Proportions based on nonmissing data; 1.2% missing data.

Although the link between diabetes and TB is widely accepted, previous studies showed differing strengths of association and significance (*4*), which could be attributed to variability in the prevalence of diabetes and TB in the population. We found a modest association between diabetes and TB disease. 

This evaluation was subject to limitations. We were not able to control for all risk factors for TB (e.g., HIV), which could have affected our odds calculations. Also, because diabetes screening is not a required part of the overseas medical examination, some persons with diabetes were probably missed, leading to an underestimation of the true prevalence of diabetes in this population. In the United States, ≈28% of persons have undiagnosed diabetes (*9*); this number may be greater among refugees with limited access to healthcare services (*10*). Because diabetes was significantly associated with TB, a differential misclassification may have occurred where there was more undiagnosed diabetes among refugees with a history of TB disease. If misclassification of diabetes status did occur, these findings are an underestimation of the actual strength of association between diabetes and TB. More research, such as testing for diabetes during overseas medical examinations would allow for a more accurate assessment.

Most state refugee health programs rescreen all refugees for TB as well as other infectious diseases (e.g., hepatitis B) at the time of arrival in the United States. Some states also test for diabetes. Our findings, along with the extensive literature associating diabetes with TB, indicate that a diagnosis of TB disease in a patient should trigger testing for diabetes to optimize treatment. In states that already screen for both diseases, further research could lead to promising innovation in collaboratively managing the 2 diseases. 
